# The concept of “Work-Life-Blending”: a systematic review

**DOI:** 10.3389/fpsyg.2023.1150707

**Published:** 2023-12-21

**Authors:** Katharina Steffens, Christine Sutter, Sandra Sülzenbrück

**Affiliations:** ^1^Institute of Traffic and Engineering Psychology, German Police University, Münster, Germany; ^2^IWP Institute for Business Psychology, FOM University of Applied Sciences, Essen, Germany

**Keywords:** systematic review, Work-Life-Blending, boundary management, Borders, domains, work, home

## Abstract

Work-Life Blending refers to the permeability and dissolution of boundaries between work and personal life, bringing these domains closer together. However, a comprehensive, holistic definition or conceptualisation of Work-Life-Blending is currently lacking. This research aims to address this gap by conducting a systematic literature review to define and clarify the concept. The primary objective is to identify the key factors and dimensions of Work-Life-Blending by reviewing the current state of research, and by offering a clear and precise conceptual framework to guide further research in developing measurable and concrete concepts. We conducted a systematic review following the PRISMA guidelines to achieve this, drawing on keyword-based searches. We searched for English or German manuscripts in the electronic databases Business Source Premier and PsycARTICLES, using keywords such as “*blending*,” “*blurring*,” “*fusion*,” “*Entgrenzung*,” “*Verschmelzung*,” “*Vermischung*,” “*boundary*,” “*border*,” or “*demarcation*” in combination with “*work*” and “*life*.” From 1,400 screened references between 2000 and 2023, we identified 302 eligible articles. After applying exclusion criteria, 51 records were retained. Employing a holistic approach, we developed a coding scheme to analyse the articles focusing on antecedents, processes, and outcomes of Work-Life-Blending. Articles were prioritized based on their impact, relevance, and data content. Our analysis revealed a diverse field, and we adopted Clark’s central concepts (2000) to categorize Work-Life Blending into four key areas: *Domains*, *Borders*, *Individual*, and *Interindividual*. Structural analysis allowed us to gain deeper insights into the multifaceted nature of the research field. Diversification was evident in studies exploring various aspects, such as the combination of dimensions (e.g., organizational and individual factors), correlations between factors (e.g., working conditions), and the introduction of new constructs (e.g., motivational processes). Our research addresses a significant knowledge gap in the field of Work-Life-Blending, making valuable contributions to the existing body of knowledge. By examining key categories and proposing an extended definition, this study provides a robust foundation for further investigations. As a result, we established a classification of the determinants. Given the high degree of diversification, we offer a comprehensive framework for future research, contributing to a deeper understanding of Work-Life Blending.

## 1 Introduction

Work-Life-Blending can be understood as the permeability and dissolution of boundaries between the domains of work and non-work, resulting in the integration and closeness of the domains. Blending boundaries between work and life affects both domains of life (the work life on the one hand and the private/home life on the other hand) and manifests in various ways. In our everyday experiences, the fusion of boundaries has become increasingly prevalent, driven by the flexibility in both spatial and temporal dimensions. Many individuals now routinely engage in flexible work arrangements, influenced significantly by technological advancements (Haeger and Lingham, 2014).

These developments give rise to novel concepts that replace traditional ones, such as work-life balance or work-life conflict, as these are no longer adequate to comprehensively address the complexities of contemporary work arrangements and the interface between work and life (Kelliher et al., 2019). Incorporating studies addressing these correlated constructs proves invaluable in pursuing our objective to identify and validate all relevant factors associated with Work-Life-Blending. By encompassing research on additional or interconnected concepts, we seek to leverage existing empirical evidence. Exploring these related constructs enhances our comprehension of the broader context and interrelationships surrounding the construct of Work-Life-Blending.

However, the blending of work and life has increased due to the exponential rise in information and communication technology and the growing frequency of remote work, especially due to the COVID-19 pandemic (Adisa et al., 2022). The effects of the pandemic on working life and, thus, on the blurring boundaries between work and life are currently determining the research focus. In particular, the focus lies on the positive and negative effects, for instance, of working from home (Jaiswal et al., 2022). de Araújo Vitória et al. (2022) provide a current review of the effects of COVID-19 on Work-Life-Blending.

Based on these developments, we argue that focusing on the construct of Work-Life-Blending is important to understand which factors and aspects must be considered to comprehensively examine the dynamics of work and personal life integration and its implications for individuals’ well-being and overall quality of life (e.g., individual preferences or contextual constraints in work or private life). Furthermore, the development of a standardized measure for Work-Life-Blending constitutes a pivotal step in comprehending the impact of this concept on workers’ health and well-being, and in formulating strategies to mitigate adverse effects. Addressing these concerns constitutes a significant aspect of our work, involving the meticulous selection, prioritization, and organization of integrated studies within the expansive and diverse field of research. Consequently, our article embraces a holistic approach to the construct, aiming to identify and consider all relevant factors and aspects.

While many relevant research approaches focus on individual subjects or distinct areas related to the blending of work and life, such as the impact of flexible working arrangements on employee performance (De Menezes and Kelliher, 2011), our research aims to join holistically driven approaches, such as the work-fusion-scale (Haeger and Lingham, 2014), the Flex-Work-Phasenmodell (Weichbrodt et al., 2014), or the work-nonwork boundary management profiles (Kossek et al., 2012). These frameworks underscore that the subject of Work-Life Blending necessitates examination from various angles and categories, incorporating diverse factors within an overarching conceptual framework. These factors span organizational aspects, structures, and processes, encompassing working life, daily work, work tasks, elements from a personal perspective, private life, and leisure time.

However, the blending of work and life is a complex phenomenon lacking a comprehensive understanding and a standardized measurement scale. Our research aims to address this gap by identifying and validating all relevant factors associated with Work-Life-Blending, with the overarching goal of establishing a consistent framework for future research and contributing to the development of a holistic measurement scale. Thus, this study aims to address the following review question: What are the key factors and dimensions of work-life blending, and how can they be integrated into a holistic concept and into a generic definition? Considering this, the aim of the present study is three-fold: (1) to review the current state of knowledge on Work-Life-Blending, (2) to define and clarify the concept of Work-Life-Blending, and (3) to outline a future research agenda in this field.

Therefore, our systematic literature search will place a specific emphasis on generic and holistic approaches. This emphasis is crucial for enhancing the measurability and comparability of the Work-Life-Blending concept. Through this approach, our goal is to conduct empirical examinations of the effects of blending, thereby establishing a cohesive foundation for future research. This strategic emphasis is pivotal in generating a consistent basis that not only enables rigorous empirical examination but also ensures comparability across studies, contributing to the development of a standardized framework for future investigations.

## 2 Theoretical framework

Research on the relationship between work and life has a long history. Initial perspectives delineated work and life as distinct entities; however, since the 1970s, scholarly inquiry has shifted towards exploring the intricate interconnections between work and life. Several periods and theoretical frameworks have shaped the evolution of Work-Life-Blending, ranging from the impact of the industrial revolution (Clark, 2000) to the advent of the digital age (Allen et al., 2015). Nevertheless, the concept of Work-Life-Blending has emerged as a response to transformative shifts in both work and life dynamics. Clark (2000), for instance, describes the development in detail and postulates a high level of complexity in the interplay between work and life. However, the theoretical basis with which positive or negative effects can be explained still needs to be included (Clark, 2000). Clark calls for a theory that includes the causes and influencing factors and the positive and negative effects to create a concept for individuals and organizations in which the positive effects predominate (Clark, 2000).

Building on existing research, the work/family border theory by Clark (2000) aims to explain how individuals deal with their different domains to gain balance. The theory describes different factors of the work/family interface (such as domains, borders/boundaries, or individuals involved). It provides insight into the different levels (such as the extent of segmentation or integration or the distinction between peripheral vs. central domain membership) that must be considered. Central concepts from the work/family border theory (Clark, 2000) are the two domains of work and home, the boundaries between work and home, and the individual differences of the individuals involved. In the subsequent sections, we will clarify the central concepts of domains, borders, individuals, and interindividual in more detail. Our objective is to provide a comprehensive and inclusive overview, emphasising the antecedents, processes, and outcomes associated with the phenomenon of Work-Life-Blending.

Concerning the two domains, a distinction is made between the domain of work and the domain of life/home. However, the separation of these domains emerged from the industrial revolution, as, for the first time, the tasks and responsibilities of work and family life had to be performed at different times and places (Clark, 2000). Processes in the perception of the different domains can be explained by differently perceived roles, thought patterns, and the actual behavior of individuals (Clark, 2000). Clark has compiled the essential characteristics for these differences: differences in valued ends and means, differences in culture, and differences in subjective management between the two worlds (Clark, 2000). Outcomes arise through the interaction of the two domains or, particularly, through the subjective handling of the boundaries (Clark, 2000). The conflict between work and life is one of these outcomes. Early research approaches identified and compiled sources for the conflicts, for example Greenhaus and Beutell (1985). They examined three major conflicts: time-based conflict, strain-based conflict, and behavior-based conflict (Greenhaus and Beutell, 1985). Studies have also shown that conflicts can go both ways and that a distinction is made between the work-to-family conflict (WFC) and the family-to-work conflict (FWC) (Frone et al., 1992). In terms of outcomes, Clark summarises various studies, describing conflicts or balance as possible outcomes (Clark, 2000). A review by Sirgy and Lee (2018) deals with an integrated conceptualisation of work-life balance. The authors state that, for instance, the segmentation effect is also a strategy for work-life balance, which means that segmentation between work and life can prevent that negative experiences in work spillover to the private life, and thus maintaining balance and satisfaction (Sirgy and Lee, 2018).

The boundaries between the domains are defined as the point where, for example, work and life separate and demarcate. Clark (2000) describes the essential determinants of these boundaries. Accordingly, boundaries are created and defined by physical, temporal, and psychological factors (Clark, 2000). The physical boundaries refer to the location where tasks are performed or the behavior of a particular domain is exhibited (e.g., in the office). The temporal limits refer to when tasks are completed (e.g., during business hours), and the psychological limits are drawn by the individual itself and are expressed (e.g., through thinking patterns, behavior patterns, and emotions) (Clark, 2000). According to Clark (2000), the key properties of boundaries are their permeability and flexibility, which can influence the processes between work and life. A high level of permeability is generated, for example, by the fact that professional interruptions occur during leisure time. The flexibility describes the possibilities to design the boundaries between the domains, such as locally through remote work. Many studies mention flexibility as an opportunity for a better work-life balance, for example, Adisa et al. (2017). However, these possibilities can also create problems. Mental stress can result from constant and permanent availability (Dettmers, 2017) and physical stress, for example, due to a lack of ergonomics in the home office (Davis et al., 2020). Regarding the inconsistent results, Kossek et al. (2023) reviewed 338 studies to organize the literature around work-life flexibility policies. The results show that previous research often only evaluates the availability of individual policies and individual outcomes, while underestimating boundary control, extent of use, policy bundling, implementation, and the multiple levels of outcomes (Kossek et al., 2023). The authors propose that future research and practice should focus on recognizing and measuring different types of employee boundary control (spatial, temporal, size-related, permeability, continuity) as crucial aspects of policy experiences, viewing the implementation of work-life flexibility policies as a process involving availability, access experiences, use, and outcomes, with consideration of stakeholders and contextual factors and developing innovative approaches to address emerging policy issues (Kossek et al., 2023). Another characteristic of borders is their blending. Following Clark, blending occurs when there is high permeability and flexibility of borders, and the domains can blur temporally, locally, or psychologically (Clark, 2000). Permeability, flexibility, and blending determine how strong or weak each boundary is (Clark, 2000). Strong borders are characterized by impermeability and, they are not flexible, so domains cannot blend (Clark, 2000). As part of our synthesis, we will clarify this definition of Work-Life-Blending and, if necessary, expand upon it.

Concerning the individual differences of individuals involved, Clark describes that the domains and even the boundaries are the results of self-production. Therefore, we take up these topics that Clark subsumes under Border-Crossers in our category *Individual* (Clark, 2000). Border-Crossers can be characterized by three factors: peripheral or central domain membership, influence, and identification (Clark, 2000). In this context, central domain membership means, for example, that a person has internalized the culture, language, and values from a domain, that the person has the necessary skills, that they are connected to other individuals in the domain, and that they are familiar with and committed to the tasks and responsibilities. Influence arises through competencies, the network with other individuals in the domain, and the internalization of the respective culture and values. Identification can be measured by how closely a person is connected to a domain’s values, roles, and responsibilities (Clark, 2000). In the context of the individual handling of the domains and the borders, the research examined different boundary management preferences, such as, on one end, the integration and, on the other end, the segmentation (Nippert-Eng, 1996). Integration and segmentation differ in their role identities. While there is a high contrast in work and home identity within a segmented role, there is a low contrast between role identities within an integrated role (Ashforth et al., 2000).

Concerning our *Interindividual* category, the foundation of Clark’s theory is “Border-keepers and other domain members” (Clark, 2000, p. 761). She posits that other individuals play a large role in how well a person can manage domains and borders. A Border-keeper is defined by the fact that the person determines and defines a domain (e.g., as a supervisor at work or partner at home). They “…negotiate what constitutes the domain and where the borders between them lie” (Clark, 2000, p. 476). Clark lists two antecedents in this context: other-domain awareness and the commitment to Border-crossers. The awareness is measured by how well, for instance, a manager knows and considers the requirements from another domain. Commitment describes the degree to which a Border-Crosser supports a person with responsibilities beyond their domain in another (Clark, 2000). In her theory, Clark focuses on various variables that influence the interaction between work and life (Clark, 2000).

Various research streams address facets of boundary blending, including the topic of borders, as exemplified by border theory (Clark, 2000), management aspects such as boundary management theory (Ashforth et al., 2000), and the interplay of work and life, as illustrated by the work-family interface (Kreiner et al., 2009). Nevertheless, individual and organizational factors are often considered separately, so no multidimensional concept summarises and considers all aspects (Kossek and Lautsch, 2012). According to Desrochers and Sargent (2004), the differences between the work/family border theory (Clark, 2000) and the boundary theory (Ashforth et al., 2000) are minor. Consequently, the current study will be grounded on one selected theory, the work/family border theory (Clark, 2000), and its central concepts of Domains, Borders, Individual, and Interindividual, as introduced above. We selected this specific theory as the foundational framework for our study to bring focus and coherence to our research. This deliberate choice allowed for thoroughly exploring the theory’s concepts. While acknowledging the inherent limitations of the chosen framework, it played a pivotal role in defining and clarifying the concept, as well as outlining a future research agenda within our study.

## 3 Methods

To achieve a comprehensive and generalized understanding of factors associated with the blending of work and life, we conducted a systematic review of pertinent literature. This involved an examination of the antecedents, processes, and outcomes of Work-Life-Blending, employing a keyword-based search methodology as outlined by Liberati et al. (2009). The blending of work and life is subject to constant change. Considering the significant amount of information published on the topic and with the understanding that current developments of the last decades have brought the topic forward, we searched from 2000 to 2023. To summarise our research topic accurately and reliably, we follow the PRISMA (Preferred Reporting Items for Systematic Reviews) guidelines for systematic reviews (Liberati et al., 2009; Moher et al., 2009; Page et al., 2021) to ensure complete and transparent reporting. To do this, we developed a search strategy that fits our research goals. In alignment with our goal to comprehensively and holistically capture the state of research and clarify the Work-Life-Blending construct, we formulated a search strategy that incorporates main keywords and their inductively derived synonyms.

We covered the main domains “Work” and “Life” as well as the “boundary” and keywords on the mechanism of blending. We searched for articles in English or German based on the following keywords: “Work”; “Arbeit”; “Family,” “Famil*”; “Life,” “Leben,” “Home,” “Nonwork,” “Blending”; “Blurring”; “Fusion”; “Entgrenzung”; “Boundary”; “Boundar*”; “Border,” “Demarcation.”

We employed combined search terms in the titles and abstracts of the search fields. We conducted searches for all manuscripts published between 2000 and 2023 in the electronic databases Business Source Premier and PsycARTICLES. The data source Business Source Premier includes 4,269 indexed, active journals and abstracts. The data source PsycARTICLES includes all APA-published scholarly journals, Journals from the Canadian Psychological Association, and the Hogrefe Publishing Group. The reason for selecting this approach is to efficiently obtain a representative overview of the generic and holistic literature. For this reason, we have also decided not to include any other databases.

**Figure 1:**
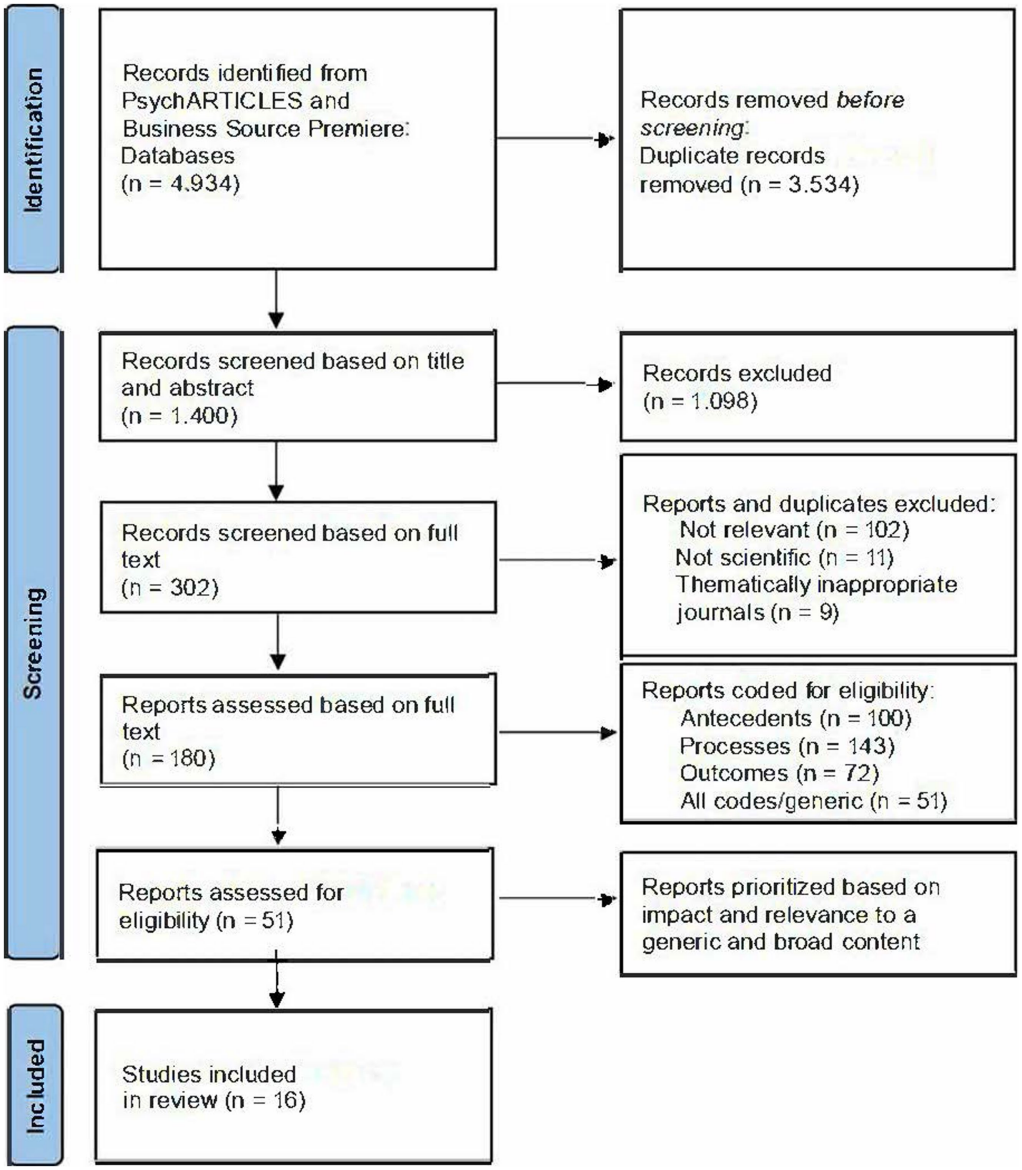
PRISMA-flowchart of the systematic literature review.

Figure 1 illustrates the PRISMA-Flowchart of the review process. The depicted process is shown below. We identified 4,934 studies through database screening. After removing duplicates, 1,400 studies remained. It is important to consider which type of study is most appropriate for answering the review questions (Petticrew and Roberts, 2006, p. 59). We established our exclusion criteria prior to the initial screening based on the titles and abstracts. Specifically, we excluded non-scientific articles such as letters, book reviews, or editorials (*Type of publication*), reports that are not published in reputable or thematically relevant journals (*Sources*), articles written in other languages than English or German (*Language*), and studies that were not located in the work/life environment (*Setting/Reference*). We applied the defined exclusion criteria and excluded 1,098 records in the first screening based on the title and abstract.

The remaining 302 studies were eligible for full-text review. During the full-text screening of the articles, our objective was to assess their eligibility for the review. To be considered, an article had to concentrate on the work/life environment - ideally with a focus on blended work/life domains. We applied the same exclusion criteria as above and excluded another 122 records after screening the full text: 102 articles were thematically irrelevant, 11 were not scientific publications, and nine were published in thematically non-relevant journals. Finally, 180 articles remained for the full-text assessment. To conduct a more in-depth analysis aligned with our research aims, we applied codes and criteria to determine the eligibility of certain characteristics in the papers.

According to the aim of the study and based on our preliminary literature review, the eligibility criteria consist of the categories of *Antecedents*, *Processes*, and *Outcomes*. The criteria of *Antecedents* were defined by the following questions: *What causes borders and blending? What leads to blending? What are the factors the boundaries (or the blending) consist of?* Respectively, the questions for the criterion *Processes* were: *What causes boundaries to blur? Which processes are involved? How do these processes evolve?* To assess the criteria *Outcomes*, we asked: *What are the results? What are the consequences?*

The coding was not exclusive, as one article may consider more than one of these areas. Of the 180 classified articles, 100 focused on antecedents, 143 on processes, 72 on outcomes, and 51 were generic. We excluded studies focusing on the extraordinary dynamics of the COVID-19 pandemic, such as mandatory work from home (Van Gelder et al., 2022), as those effects were outside the aim of the present study.

In the final phase, we focussed on the 51 generic articles. These articles met all three specified criteria: antecedents, processes, and outcomes. To prioritize the articles according to their impact and relevance concerning the generic and broad content, three raters (the first author and two other researchers) independently categorized the articles using the categories of *A*, *B,* and *C*.

Category *A* represents the articles with the highest relevance and impact according to our aim. These articles were considered the best fit for the review. They were included for further analysis as they provide a comprehensive understanding of the research topic and factors related to the blending of work and life. Category *B* represented articles with a moderate level of relevance and impact. The articles were, therefore, not included. Category *C* includes articles with the lowest relevance and impact. They have minimal relevance or do not significantly contribute to the aim of our study. Articles in this category are excluded from the review.

The interrater reliability was 43%. For the final categorization, the three raters discussed their categorization of each article until they found a common consensus on their prioritization. Finally, our qualitative synthesis included 16 studies (see supplementary material) with original research data.

## 4 Results

Regarding our research aims to (1) review the state of the art of Work-Life-Blending, to (2) define and clarify the concept, and (3) to outline a future agenda, we have compiled our results below. The results section will be structured according to the categories of *Domains, Borders, Individual,* and *Interindividual*. With the detailed presentation of the research results, we will prioritize addressing the first research goal. Since the review of the state of research is a prerequisite to address research aims (2) and (3), we will address all goals in the discussion section of the paper.

### 4.1 Domains

The domains work and life refer to the separate areas of an individual’s life, including different rules, thought patterns, and behavior (Clark, 2000). We will present five studies that provided relevant input concerning the issue of domains. The articles explore the interconnectedness of work and life, examining how interactions between these domains impact personal and organizational issues. In the context of our research on blending boundaries, we will specifically focus on the two domains: work and life.

In a multi-wave survey with *N* = 251 financial sales professionals, Lapierre et al. (2016) examined whether involuntarily working more from home (teleworking) is related to higher time-based and strain-based work-to-family conflict (WFC), considering employees’ boundary management strategy (integration vs. segmentation) and work-family balance self-efficacy as moderators. Results showed that involuntary work from home was related to a higher strain-based WFC but not to a higher time-based WFC. While no moderating effect could be determined for the boundary management strategy (such as integration or segmentation), the accomplished moderator analyses showed that the positive association between the time or strain-based conflict and the involuntarily work from home are stronger, when individuals have a weaker self-efficacy in balancing work and family.

The study by Piszczek (2017) addresses information and communication technology such as smartphones etc. The study examined the conditions (such as after-hours electronic communication expectations or segmentation preferences) under which work-family technology use is associated with greater boundary control and thus leads to job resources or job demands (e.g., emotional exhaustion). Therefore, he conducted a multi-wave online survey with *N* = 163 alums of a human resource management master’s degree program. Overall, the study shows that using technology can lead to different outcomes. For employees who prefer role integration, the technology use is associated with higher boundary control. For employees who prefer role segmentation, the technology use is associated with lower boundary control (Piszczek, 2017). Furthermore, findings show that the expected after-hours electronic communication can force work-family technology use despite individual preferences and that boundary control is negatively linked to emotional exhaustion (Piszczek, 2017).

The article by Leppäkumpu and Sivunen (2023) examined how communication between different domains (across-the-border (ATB) communication) can be used to manage work-life boundaries. The article explores the interconnectedness of work and life domains, how communication practices and strategies can influence the blending or separation of these domains, and how individuals can navigate and maintain boundaries between work and life through communication techniques in various contexts. They interviewed *N* = 32 participants, comprising journalists (*N* = 16) and their relational others (*N* = 16). They found that communication across-the-borders generates a shared understanding between both persons that helps to deal with the responsibilities of both domains. They found two types of support through communication: instrumental and emotional. Overall, the article states that communication plays a relevant role in managing the boundaries between work and life and that clear and open communication in various domains can develop effective strategies to lead to positive outcomes (Leppäkumpu and Sivunen, 2023).

Spieler et al. (2017) examined the effects of the use of flexible working hours on the blending of work and life in two studies. In particular, they investigated whether the relationship between the use of flextime (flexible working time arrangements) and affective well-being is mediated by the level of goal completion and the personal boundaries around one’s work and life domains, such as boundary strength. In study 1, they conducted an experience-sampling study with *N* = 150 bank employees. Study 2 aimed to replicate the first study’s findings with a more heterogeneous sample. In the second study, *N* = 608 employees from different organizations and with different flextime arrangements participated. Their results show that flextime use was associated with stronger boundaries at home (in both studies) and stronger boundaries at work (in study 2). In addition, they found that stronger boundaries were positively associated with affective well-being, both in the same evening and the next day. In their second study, the results showed that day-specific nonwork goal completion mediated the positive association between daily flextime use and boundary strength at work. Based on these findings, they postulated that, on the one hand, chronic flextime use undermined the completion of work goals and, on the other hand, that flextime has some benefits when used occasionally but loses its benefits when used continuously. Therefore, a distinction should be made between general flextime policies and different flextime availability levels due to different outcomes (Spieler et al., 2017).

The article by Jostell and Hemlin (2018) examined how mobile working (working from home) outside of regular working hours and individual boundary management affects the conflict between work and life. They conducted a cross-sectional online survey with *N* = 71 full-time employees at the headquarters of a multinational high-tech firm. The study investigated both directions of conflict - work interrupts family (WFI) and family interrupts work (FIW). They found no relationship between the number of teleworking hours and work-to-family conflict (WFC). In the investigations on permeability, however, the study underlines previous research results: more permeable boundaries and interruptions in leisure time due to work led to a higher level of WFC. This finding indicates that teleworking after hours does not primarily lead to conflicts between work and life. Rather, these conflicts arise due to breaks in leisure time caused by work (Jostell and Hemlin, 2018).

Taken together, the articles provided in the category *Domains* focus on technology usage, communication practices, flexible working hours, mobile working, and boundary management strategies. Several key themes and connections become clear across the studies, providing relevant insights. The findings are based on the idea that work and personal life are like different parts of a person’s life. Each part follows its own rules, ways of thinking, and behaviors (Clark, 2000).

### 4.2 Borders

Secondly, we explored the category of borders. In this context, our specific focus lies on the properties of the borders. Borders are the lines that demarcate the domains of work and home, which can be physical, temporal, and psychological. These borders are not fixed but are dynamic and permeable and can be affected by contextual and individual factors (Clark, 2000). A total of six of the included articles are dedicated to exploring the research area of borders, which, as previously mentioned, we believe effectively addresses our primary research objective.

McCloskey (2013) examined the characteristics of employees with different boundary types and whether these individuals experience different levels of work–family conflict as well as job and life satisfaction. They conducted an online survey with N = 65 undergraduate and graduate alums. They differentiated between various combinations (general/work or home boundary) and configurations (flexible/permeable) of boundaries to investigate the processing of the work/life conflict, balance, or satisfaction of work and life for employees. Specifically, they examined the different characteristics of boundary types. They describe that the concepts of permeability and flexibility are often used simultaneously in research and postulate that both properties of boundaries can also occur separately and independently of one another. In addition, they postulate that both permeability and flexibility can be viewed from two directions - work and home. The study shows that the flexibility and permeability between work and life can lead to different results. It proposes new approaches for further research, in which the properties of boundaries - permeability and flexibility - are viewed as different constructs, and the effects of the blending between work and life can be very diverse. In total, they postulate eight different boundary configurations that deal with the combinations of boundary flexibility on the one hand and with the work/home permeability on the other hand, such as “inflexible, home permeable, work not permeable” (McCloskey, 2013, p. 56), what means that work occurs during a fixed schedule without dealing with personal issues in the meantime. Therefore, individuals must consciously pay attention to a correct (most appropriate for individuals to maximize comfort and success) configuration to minimize conflicts and gain job and life satisfaction. “Understanding and consciously choosing to right boundary configuration is a critical area of inquiry in a mobile world.” (McCloskey, 2013, p. 68).

Based on a survey with a matched set of *N* = 503 subordinates and their spouses, Ferguson et al. (2015) examined how supervisor instrumental support and organizational segmentation support can contribute to flexibility regarding the boundaries between work and life. In addition, the study examined the effects of flexibility on employees and their spouses. The purpose of the study was to extend the research of boundary management by focusing on supervisor instrumental support and organizational segmentation support as critical resources for employees’ work boundary flexibility. The study finds that supervisor instrumental support and organizational segmentation support are working conditions that make boundaries more flexible and thus provide resources to the employees on the experience of family functioning and their affective commitment. In addition, these effects also affect other individuals in the social environment of the employees, for example, the spouses’ commitment to the incumbents’ organization (Ferguson et al., 2015).

Hyland and Prottas (2017) examine how the flexibility and permeability of the boundaries between work and life affect the spillover between work and life domains. Based on an online survey with *N* = 4.392 business school alums of a private university, Hyland and Prottas examined two boundaries such as the work boundary and the home boundary in both directions, and analyzed flexibility and permeability by using directional and dichotomous measures (home to work and work to home) of both negative and positive spillover. The research provides differentiated results about the effects of flexibility and permeability in boundaries. Flexibility can reduce time- and strain-based stress in both directions, from work to leisure and leisure to work. Flexibility also creates a positive spillover from private life to work. A positive correlation to time-based spillover was found for permeability from work to private life and from private life to work. The study also found that the permeability between the domains of life is asymmetrical: the relationship between the permeability of the work boundary and the spillover from work to home is stronger than that between the permeability of the home boundary and the spillover from home to work. The study’s findings suggest that the boundaries of work and life should be viewed separately. Depending on the directions, the boundaries can differ in flexibility and permeability, are perceived differently, and, accordingly, lead to different outcomes. The authors propose that the flexibility of private life, in particular, has not yet been researched well enough. In the context of increasing digitisation, the authors describe the relevance of flexibility and permeability of the boundaries between work and life for organizations as well as for employees, suggesting an optimal level of integration. Overall, the article illustrates that understanding flexibility and permeability in the context of the blending of work and home/life must be viewed from different perspectives/directions, as these can influence the corresponding effects and the degree of spillover.

Eddleston et al. (2017) investigated the physical integration of work and life in two studies. While a theoretical model was proposed based on the first qualitative study, the proposed model was tested quantitatively in the second study. Therefore they conducted a survey with *N* = 299 remote workers. Specifically, they examined the advantages and disadvantages of working exclusively from home, the effects of remote work on the transition between different roles, the different ways men and women deal with the demands of work and life, as well as the influence on the FWC (Family-to-work conflict) and WFC (Work-to-family conflict). The authors examine how remote employees deal with the boundaries between the domains of life. Even if the workplace physically exists in the private sphere, there are different degrees of integration or segmentation of work and life. Research shows that within the physical integration of remote work, new temporal or tangible boundaries are created to segment the two areas of life still (Eddleston et al., 2017). Their findings clarify the various levels and interfaces that result from the physical integration of work into the home/life. There are aspects such as the extent, the circumstances, and the individual or gender-specific boundary management strategy (because of the uniquely experienced boundaries between work and home by men and women) that must be taken into account to consider possible effects and outcomes. Their findings also indicate that general statements about remote work on the effects of flexibility on the degree of integration and potential conflicts and risks are not possible.

The study by Kim and Hollensbe (2017) examines individual and situational factors as antecedents of work boundary permeability, considering both negative and positive effects. They conducted two survey studies with *N* = 308 full-time employees working in the information technology sector. Based on previous empirical findings, Kim and Hollensbe (2017) examine the factors of *segmentation preference*, *workload*, and *home demands* as antecedents of permeability. Their findings show that the permeability of the *work boundary* leads to time- and load-based stress but not to positive (affective and instrumental) spillover. Therefore, they state that the boundary permeability direction (work to home or home to work) is an important aspect. They suggest a distinction should be made between the domain from which the permeability is viewed, and which leads to spillover effects in another domain. These are similar findings to those of Hyland and Prottas (2017). The permeability of the work (work domain is permeated by home-related matters) or home boundary (home domain is permeated by work-related matters) can be different. The findings of this article also indicate that further research on the antecedents and consequences of the boundary configuration permeability is necessary since there is still no conclusive research as to whether the effects of permeability are functional or dysfunctional (Kim and Hollensbe, 2017).

A second article by Kim and Hollensbe (2018) examined the impact of technology-related pressure on home boundary permeability, the positive and negative consequences of home boundary permeability, and the moderating role of home support. They conducted a study with *N* = 267 full-time employees in the Midwestern United States. Their study results show that individual preferences regarding segmentation and situational factors, such as workload, can lead to boundary permeability. In addition, they found that a high degree of permeability of the work boundaries leads to a greater time- and stress-related conflict but not to an affective and instrumental positive spillover. The permeability of the boundaries between work and life is always linked to a direction. This means that two directions of permeability must always be considered independently of one another, for example, as mentioned before, the permeability of the work boundary or the home boundary. To this end, research must provide more in-depth knowledge in the future, particularly on the antecedents and outcomes of permeability. There is still no unified state of research about which functional or dysfunctional effects can result from high permeability (Kim and Hollensbe, 2018).

The studies show that the borders demarcate the domains of work and home and can be physical, temporal, or psychological (Clark, 2000). The findings underscore the dynamic and permeable nature of these borders, influenced by various contextual and individual factors.

### 4.3 Individual

With the first two categories, we compiled research findings on the Domains and the Borders of these domains. Some of these studies have already provided insights into the individual management of demarcation. Therefore, we closely examine individual perspectives and summarise the state of the literature in this regard. As mentioned above, the category *Individual* refers to an individual’s characteristics, including personal identification with the domains and the influence, considering individuals competencies and the affiliation with central members of the domains, as well as the internalized culture and values of the domain (Clark, 2000). We identified four studies that provide further research insights into our category. Additionally, this compilation contributes to our first research objective, complementing the existing findings.

To understand the strategies of individuals in dealing with the boundaries of work and life, the study by Wright et al. (2015) explores the individual ways of dealing with the boundaries of work and life and the conflict between work and life of men and women. The study is set up as evaluation research and utilises the WorkLife Indicator™ (WLI) to record the individual boundary management strategies. Data were collected via the initiative use of the WLI and by including product sales. *N* = 1.800 professional workers participated. The WorkLife Indicator is a tool to measure the differences between individuals how they deal with the integration or separation of the domains of life. It measures three aspects, (1) the degree to which individuals combine or separate behavior between work and family (two dimensions are possible: Family Interrupts Work and Work Interrupts Family), (2) the degree to which individuals identify with and invest in roles (two dimensions: work focused and family focused) and (3) the degree to which individuals feel in control of how they manage the boundaries between work and family (Hannum et al., 2011). The study aimed to evaluate the measurement equivalence/invariance of the WLI across gender and to examine whether the WLI scores exhibit predictive invariance concerning important work-life outcomes. Considering our research aim, we focus on the results concerning gender differences. The study found that women and men have different views on managing work-life boundaries, such as “women and men view work interfering with nonwork and family-centred identity differently, which is not surprising given that woman are expected to be more involved in the family than work, while men are expected to be more involved in work than family.” (Wright et al., 2015, p. 145). In addition, the results indicated, “that women are more likely to experience negative outcomes when work interferes with nonwork, while men are more likely to experience negative outcomes when nonwork interferes with work.” (Wright et al., 2015, p. 145). From this pattern of results, the authors derive various topics that should be considered in future research, such as demographic variables like relationship status, organization policies, country culture, and amount of family responsibilities. In addition, the authors suggest that research should look beyond demographic variables into the causes of different boundary management strategies.

The aim of the study by Foucreault et al. (2018) was twofold. They aimed to examine whether an organizational culture of integration can influence the ability to act on personal preference for segmentation. They also examined whether a mismatch between culture and individual preference could influence the emotional state of individuals and, if so, how. The study included *N* = 243 employees. In detail, the results showed that emotional exhaustion was negatively associated with psychological detachment from work. The results also revealed that psychological detachment was negatively associated with the individuals’ perception of an organizational culture of integration and positively associated with the individuals’ preference for segmentation. Furthermore, the study confirmed a positive association between a preference for segmentation and emotional exhaustion. These results indicate that the organizational culture can change individuals’ boundary management, which can have relevant consequences for employees’ possibility to detach and relax from work in their free time. These findings indicate that there are very strong connections between individual and organizational factors. Overall, external factors (organizational but also factors from the family environment) have a strong influence on the individuals’ options to act in line with personal preferences for segmentation. The study proposes that the influence of organizational culture needs to be further researched and measured. In addition, it should be investigated what most influences how employees deal with their boundaries (Foucreault et al., 2018).

Based on Self-Determination Theory (Deci et al., 2017) and Boundary Theory (Ashforth et al., 2000), Peters and Blomme (2019) examined how flexible workplace designs can trigger multiple motivational processes underlying gendered work/nonwork integration behaviors and how these behaviors affect work/life conflict. They conducted a theoretical elaboration to present scenarios on how flexible workplace designs can trigger multiple motivational processes underlying gendered work/nonwork integration behaviors, and how these affect work/life conflict. The approach posits that fewer boundaries do not mean the conflict will become less. Fewer boundaries indicate that employees have a higher workload to coordinate tasks with others and find new strategies to manage work and home-related tasks. It must be considered that more flexibility and permeability can also generate more pressure, for instance, by being forced to be available or to respond to work-related topics in leisure time. The authors point out that long-term studies are necessary to precisely analyse the opportunities of flexible workplace design for all stakeholders.

The study by Lachance-Grzela et al. (2022) aims to find out if there are different profiles of employees according to their work motivation, role blurring, and psychological well-being. They conducted a survey with *N* = 200 currently employed adults. They found five profiles of workers, including the labeled roles of “Reluctant,” “Autonomous” or “Highly Motivated” (Lachance-Grzela et al., 2022). The study results show that varying levels of role blurring can coexist with varying levels of well-being. It could not be found that a high role blurring is associated with low well-being. This study thus confirms existing theories, such as Boundary Theory (Ashforth et al., 2000) and Border Theory (Clark, 2000). It states that different aspects, such as individual characteristics or contextual factors, influence employees’ well-being in managing the boundaries between work and life. Also, it states that these aspects also can interact with each other, for example, individual’s role blurring resulting from high job demands outside of work hours can lead to varying effects on well-being, depending on the personal preference for segmentation or integration (Lachance-Grzela et al., 2022).

The studies in this section explore individual boundary management strategies, the influence of organizational culture on boundary preferences and emotional state, the impact of flexible workplace designs on motivational processes, and the relationship between work motivation, role blurring, and psychological well-being.

### 4.4 Interindividual

According to Clark (2000), other individuals play a significant role in an individual’s ability to manage different domains and establish boundaries between them effectively. A Border-keeper, such as a supervisor or partner, is responsible for defining and determining a specific domain and negotiating its borders (Clark, 2000). While the other categories of work-life blending, such as domains, borders, and individual, also play significant roles, the interindividual category emphasises the social connections and dynamics that arise when individuals navigate the complexities of combining work and life. We identified one study, which focuses on the interactions and relationships between individuals in the context of their work and personal lives and therefore provides research insights into our category. This finding helps us complete our overview of the state of the art in relation to our research objective.

The study by Wan et al. (2022) explores the preferences of dual-earner couples for boundary segmentation between work and family, and its impact on work–family conflict. In a survey study of N = 161 dual-earner couples, they investigate the interaction between partners’ preferences. The findings reveal that couples may have different preferences, and different preferences are associated with higher conflict levels (Wan et al., 2022). The study shows that it is relevant for both organizations and individuals to understand the impact of differing preferences, such as between oneself and the partner, and give recommendations for organizations to offer trainings or workshops to educate their employees and underline the interindividual dynamics (Wan et al., 2022).

By exploring the preferences of dual-earner couples for boundary segmentation and its impact on work–family conflict, the study highlights the importance of interpersonal dynamics and communication in Work-Life-Blending. The key themes include preferences for boundary segmentation, the impact on work–family conflict, interindividual dynamics, and organizational relevance.

## 5 Discussion

The main objective of our study was to contribute to a deeper understanding of Work-Life-Blending by reviewing existing knowledge, defining the concept, and identifying relevant factors. We pursued three aims: (1) to review the state of the art about the delimitation of work and life; (2) to define and clarify the concept of Work-Life-Blending; and (3) to develop a future agenda for further research on Work-Life-Blending.

Primarily, the notion that work and personal life are completely separate domains might oversimplify the intricate realities of modern existence. This becomes more apparent when considering the evolving nature of work and the substantial impact of technology. In this context, focusing on how technology affects how individuals handle boundaries brings up new perspectives, for example, when work can be anywhere and anytime, personal preferences and strategies, as well as self-efficacy regarding work-life-balance, need to be stronger. Our study investigated how individuals manage their work-life boundaries, including when they work from home, considering personal and situational factors. It becomes clear that effectively handling these boundaries is crucial for positive effects. We want to underline the increasing integration of work and personal life in today’s world, with flexible and permeable borders. However, our closer look reveals that while these boundaries become more flexible and permeable due to evolving work environments, individuals are also shaping distinct boundaries based on their individual preferences. This shows the new and important role of personal responsibility and self-management.

Moreover, it is crucial to assess where differences lie, such as in the utilization of flexible work options, support systems, or management strategies. Do certain individuals have advantages due to their job, relationships, or gender? Highlighting these differences is essential for organizations to ensure that a positive work-life interaction is fair and equal for everyone. From our perspective, this could have significant practical implications for improving working conditions.

Furthermore, the idea of new boundaries emerging due to changes in how we work requires a closer look with more details. We propose a more detailed exploration to gain a clearer understanding of these boundaries and how their configurations impact the blending of work and personal life, as well as overall well-being.

Based on our results, we have identified several periods with different focuses that contribute our research. The first period concentrated on exploring the different types of boundaries between work and home, including physical, temporal, and psychological boundaries. Research during this period examined various characteristics of these boundaries, the role of supervisor and organizational support, the flexibility and permeability of boundaries, and the physical integration of work and life (e.g., McCloskey, 2013; Ferguson et al., 2015; Hyland and Prottas, 2017). The second period encompassed the examination of external factors, such as organizational and family environments, and their impact on individuals’ ability to manage work-life boundaries (e.g., Wright et al., 2015; Eddleston et al., 2017; Foucreault et al., 2018). The third period focused on understanding the relationship between work and life domains, exploring how individuals balance and manage boundaries between the two (e.g., Lapierre et al., 2016; Piszczek, 2017; Spieler et al., 2017; Jostell and Hemlin, 2018).

The insights highlight the interplay of individual, organizational, and societal factors, facilitating the development of a consistent framework and a holistic measurement scale in this area. In synthesizing periods, it becomes evident that the research has evolved from understanding boundary types and characteristics to investigating the impact of internal and external factors and exploring the nuanced interactions between work and life domains. In this context, it also can be stated that the research field is becoming more and more diversified, for instance, the properties of boundaries could be seen as different constructs (McCloskey, 2013). In many research approaches, dimensions are combined, including organizational and individual factors, such as remote work considering individuals’ preferred boundary management strategy (Lapierre et al., 2016). Recent research focuses on the relationships between these factors and introduces other constructs into the research, for example, motivational processes (Peters and Blomme, 2019).

However, the categories of *Domains*, *Borders*, *Individual,* and *Interindividual* have proven useful as a basis for exploring the concept. They provide a valid frame on which the complexity of the research topic can be reduced. The interconnectedness of domains, borders, individual experiences, and interindividual dynamics calls for a multifaceted and adaptable approach to the concept of Work-Life-Blending. This is an essential building block for our aim to condense the state of research.

### 5.1 Review of the state of the art about the blending of work and life

Within the results presented across the categories of *Domains*, *Borders*, *Individual*, and *Interindividual*, our primary focus has been to address the first research objective - evaluating the current state of research. We will now synthesize these findings in this section. In the following sections, we will engage in discussions that relate the results to our subsequent research aims: defining and clarifying the concept and outlining a future research agenda. Through a comprehensive assessment of the main findings, their evidence and implications, as well as acknowledging the limitations, we aim to enrich the overall quality of our review.

Prior to delving into detailed discussions, we aim to provide a succinct overview of the general results: in synthesizing the literature, our emphasis was on research outcomes that encompassed holistic content. We employed coding methods to select studies for inclusion in the review, specifically prioritizing articles that satisfied all three criteria - antecedents, processes, and outcomes. Based on the theory of Clark (2000), we assumed the following categories as dimensions of Work-Life-Blending, *Domains*, *Borders*, *Individual*, and *Interindividual*. This enabled us to structure the research results and conduct a systematic analysis. In the further course of our investigation, we assigned the review results to the categories.

Incorporating all categories, our review encompasses a total of 16 studies. In pursuit of our primary objectives and the goal of identifying factors associated with Work-Life-Blending, our research has made a significant contribution. Through the summary of key topics and connections derived from the presented studies, we aim to offer a comprehensive understanding of our main findings, shedding light on the intricacies inherent in the blending of work and life.

The research field of the blending of work and life is extensive and constantly evolving. In particular, the COVID-19 pandemic has unexpectedly and quickly changed the interface between work and life (de Araújo Vitória et al., 2022). For example, the personal preference for segmentation between work and life (Kim and Hollensbe, 2017) no longer played a role in the national lockdown, as working from home became mandatory (Van Gelder et al., 2022). So, in research, the connections, especially to the antecedents, the processes, and the outcomes on the topic, are becoming increasingly complex.

However, as mentioned earlier, research needs to take a fundamental look at the overall mechanisms of the blending of boundaries, regardless of the external influences and the unexpected dynamics in the research field. Drawing from our theoretical foundation provided by Clark (2000), we will now summarise the results of our synthesis, addressing our first research objective, which is to present the current state of knowledge.

Concerning the domains, up to this day, the basis for the blending of boundaries is that work and life are understood as two domains and that nothing has changed in the scientific debate. This supports the theory of Clark (2000) stated that individuals created domains, associating them with different “*rules*,” “*thought patterns*,” and “*behavior*” (Clark, 2000, p. 753). However, the results show that there are organizational influencing factors such as the use of technologies (Piszczek, 2017), more flexible working hours (Spieler et al., 2017), and increasing telework (Jostell and Hemlin, 2018), which are changing the rules in the organizational domain, for example. There is also support for the claim that subjective perception and individual handling usually play a critical role. Here, in-depth investigations are carried out into the conditions under which positive results (e.g., flexibility or boundary control), can be achieved on an organizational and individual level (e.g., Piszczek, 2017). Recent research approaches also deal with the consistency of the blending: Spieler et al. (2017) confirmed that there are daily fluctuations, for example, how the blending of work and life can be perceived. Consistency is a new aspect that complements previous approaches and is therefore interesting for future research. Taken together, the articles grouped under the *Domains* category offer a detailed exploration of various aspects, including technology usage, communication practices, flexible working hours, mobile working, and boundary management strategies. These studies encompass a wide range of work contexts, providing insights into contemporary life and work environments and their impact on Work-Life Blending.

Regarding the concept of borders, it is evident that the delineations between work and life domains continue to hold significance in research. Clark (2000) mentioned three main forms, physical borders, temporal borders, and psychological borders. Based on these forms, our synthesis shows that there are different types and properties to investigate, such as the different characteristics of boundary types or the fact that permeability and flexibility can be viewed from two directions (McCloskey, 2013). From this, we also assume that not only the permeability and flexibility of the boundaries must be further determined but also the mutual interactions of these properties and the directions (from work to family and from family to work) from which these can be viewed. There are also research approaches that investigate various factors influencing the borders. These influencing factors, such as technology-related pressure (Kim and Hollensbe, 2018) or supervisor instrumental support and organizational segmentation support (Ferguson et al., 2015), are purposed to lead to different individual perceptions and thus outcomes, for example, commitment (Ferguson et al., 2015). Taken together, the studies categorized under “Borders” provide a nuanced understanding of the dynamic and permeable nature of work and personal life boundaries. The studies explore the configurations of two directions, flexibility, and permeability of these boundaries, shedding light on how contextual and individual factors impact their management.

Following Clark (2000), who introduced the term *Border-crossers* (with three characteristics: peripheral or central domain membership, influence, and identification), we derived our category *Individual*. Concerning this category, our research results reveal how individuals deal with the boundaries between work and life plays an increasingly important role. An interesting approach by Peters and Blomme (2019) results from integrating the self-determination theory (Deci et al., 2017) into the context of blending. Here, the individual handling of the borders is gaining importance. Due to the blending of borders, structures, and rules, individuals need to develop new structures and their own rules, utilizing self-regulation processes with their drive and motivation to achieve results. This leads to new, subjective requirements and requires new strategies. The study by Foucreault et al. (2018) appropriately shows the importance of distinguishing between personal preferences concerning the management of boundaries and the actual behaviors to manage these boundaries. To Foucreault et al. (2018), this behavior is influenced by the organizational context in which the person interacts. The influence of *organizational cultures* should therefore be further investigated in the future. Taken together, the studies in the *Individual* category provide a deep dive into how individual characteristics, preferences, and motivational processes shape boundary management strategies.

According to Clark (2000), the involvement of others, like supervisors or partners functioning as “Border-keepers,” significantly influences an individual’s ability to manage various aspects of life and define domain boundaries (Clark, 2000). While the domains, borders, and individual aspects are significant, the *Interindividual* category accentuates social connections and dynamics that arise during the blending of work and life. The study by Wan et al. (2022) delves into the preferences of dual-earner couples in segmenting boundaries between work and family, and its impact on work–family conflict. They uncover interactions between partners’ preferences, revealing potential conflicts arising from different preferences. The study emphasises the importance of grasping differing preferences for both organizations and individuals and the importance of interindividual connections and interactions.

### 5.2 Definition and clarification of the concept of work-life-blending

Building upon the existing research and taking into account our findings, our current intention is to define and clarify the concept of the blending of boundaries as comprehensively and generically as possible. Hence, we initiated this process with the provided definition by Clark (2000), which defines blending as a form of high permeability and flexibility of the borders that causes the domains to blur temporally, locally, or psychologically (Clark, 2000).

As part of our synthesis, we systematically reviewed the research results for additional conceptual or operational definitions of Work-Life-Blending to extend the definition given by Clark (2000). None of the included articles provided additional definitions for the concept of Work-Life-Blending. However, Kim and Hollensbe (2018) introduced a further aspect. They postulated that individuals who prefer integrating work and life would blend their identities between the work and home domains (Kim and Hollensbe, 2018). This aspect complements Clark’s definition, as it takes up the individual influence of one’s preferences (segmentation or integration).

Therefore, we propose extending the definition to incorporate this aspect as follows: blending can be defined as a temporal, local, or psychological blurring of domains caused by high permeability and flexibility of the borders and the personal preference to integrate the work and life domain.

**Figure 2:**
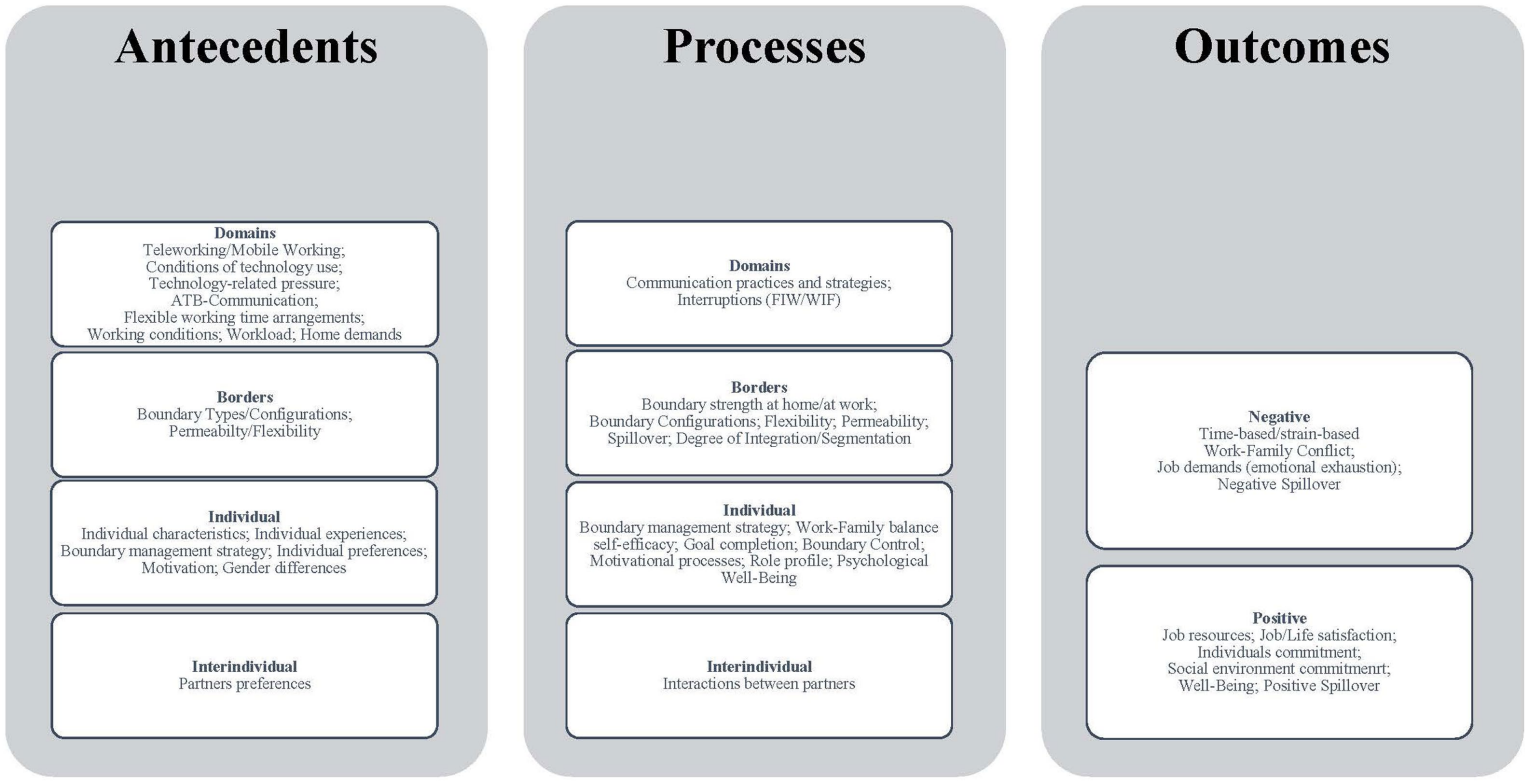
Classification of determinants.

Drawing upon our review and insights, we found that developing a classification of the determinants (see Figure 2) was a highly effective approach to managing complexity. This classification serves as a robust framework, offering a structured foundation with detailed factors, and it aligns well with our research objectives. Specifically, our aim was to comprehensively identify the key factors and dimensions of Work-Life-Blending. In alignment with Clark’s theory (2000), we generated categories to enhance the organization of our findings, facilitating a more comprehensive understanding of the subject. This approach has proven to be instrumental in achieving our research goals.

Using these categories, along with the elements of Antecedents, Processes, and Outcomes, we constructed a classification to provide an overview of the antecedents, processes, and outcomes of the construct of Work-Life-Blending. Importantly, this compilation offers insight into how the construct comes together, refraining from implying specific hierarchies or mechanisms. More detailed, we aim to generate an overview of the factors that induce the blending of work and life - identifying the necessary foundations or antecedents, exploring the associated processes that strengthen or weaken the blending of boundaries, and elucidating the potential effects that can ensue from this phenomenon.

In part, factors such as the ‘boundary management strategy” or “boundary configurations” function, are both the basis for the blending of work and life and an integral part of this process. These factors not only represent a possible basis for the blending of boundaries between the spheres, but they are also actively involved in how this dissolution of boundaries is managed and controlled. The blending between these categories is, therefore, not always distinct. For the outcomes, we decided to subdivide them into positive and negative. In this context, we find it interesting that the effects can be very far-reaching, as far as influencing the commitment of the partner.

This compilation underscores that the concept of Work-Life-Blending is a multidimensional construct, encompassing various dimensions, aspects, or facets. These dimensions are interconnected and collectively contribute to a holistic view of the construct. In particular, the individual perspective, preferences, and strategies for dealing with the dissolution of boundaries should be emphasized. We assume that the degree of dissolution is not decisive, but how the individual fit and individual perception is. From our point of view, this should be taken into account, especially in studies on the measurability of the construct. It should also be considered that individuals draw new boundaries for themselves based on their own preferences, where formal, spatial, temporal, or psychological boundaries dissolve.

### 5.3 Future agenda for research on work-life-blending

Another key objective was to delineate a future research agenda. In our perspective, this arises directly from our prior findings, as the systematic review shows that the results must be further investigated and evaluated. In relation to our research objective, we have identified and categorized significant factors. In the next step, it is crucial to examine the overarching connections. A full synthesis of the entire research field is scientifically very interesting but cannot be carried out due to the time restrictions of the research project.

Concerning the future agenda, it is crucial to join and synthesize the various research priorities, especially focusing on the antecedences, processes, and outcomes of the border concept as well as on the connections between boundary configurations, organizational and individual settings, and other influencing factors. These connections could define the Work-Life-Blending and enable a holistic definition and concept clarification. This requires further research and analysis.

Therefore, we call for future research to expand upon the valuable insights presented. This entails exploring boundary configurations in diverse work contexts and evaluating the influence of supervisor and organizational support on boundary flexibility. Additionally, while the positive effects of flextime or remote work on boundary strength and well-being are apparent, it is imperative to investigate potential challenges, such as their impact on job performance and team coordination when used continuously or involuntarily. In addition, the significance of permeable boundaries in addressing work–family conflicts is acknowledged, yet other contributing factors like family demands and workplace support structures warrant additional examination.

Furthermore, there is a need to further investigate gender differences and other demographic factors that influence individual boundary preferences. Understanding the underlying reasons behind diverse boundary management strategies can offer deeper insights into effective Work-Life-Blending approaches. Moreover, it is crucial to investigate the influence of organizational culture on boundary preferences and its connection to emotional states in the context of Work-Life-Blending. By identifying effective interventions and strategies, organizations can better support their employees in navigating work-life boundaries successfully. Following the approaches by Wright et al. (2015), topics such as demographics, relationship status, job level, social contacts, etc., should also be considered. It is worth noting that a more inclusive investigation is warranted to capture the diverse experiences of individuals in various circumstances. From our perspective, in a dynamic landscape characterized by evolving gender roles and flatter organizational structures, the interindividual influence becomes even more significant.

As previously mentioned, particularly due to the pandemic, the theme of dissolving boundaries between work and life has evolved and achieved unexpected dynamics in recent years (de Araújo Vitória et al., 2022). For example, the pandemic has led to a significant flexibilisation of work locations and remote or mobile work has been adopted and has many different and complex effects on the interface between work and life (de Araújo Vitória et al., 2022). In response to that, resource-oriented approaches should be pursued and researched. Important questions in this context revolve around what strengthens individuals and which factors contribute to an individual’s health, performance, and satisfaction in both work and life/home domains. This underscores the continued prioritization of the objective to clarify the concept.

Our research sheds light on how individuals navigate boundaries when working remotely and how personal and situational factors influence these boundaries. The primary message is that effectively managing these boundaries is crucial for achieving a balance between work and personal life in the contemporary work environment. As the borders between work and life blur, it is essential to acknowledge the rise of new boundaries.

However, there are further gaps that warrant closer examination. This includes understanding how individuals take charge of shaping their boundaries in a changing work landscape, assessing the long-term consequences of flexible work arrangements, considering the intersections of factors like gender and culture, examining the role of technology in blurring boundaries, investigating cross-cultural variations, tracking the dynamic nature of boundaries over life stages, and delving deeper into how personal values influence boundary decisions. Addressing these aspects will offer a more comprehensive understanding of modern work-life integration and guide informed strategies for enhancing individual well-being.

Furthermore, research should explore the consequences of companies transferring the responsibility of boundary setting to their employees. Does this transfer pose a risk of overwhelming employees who now need to not only fulfil their duties but also define their own limits?

Based on that, we believe it is important to make the concept of Work-Life-Blending measurable and concrete. We believe that further research can succeed in uncovering possible connections between factors and processes and their effects and give practical recommendations for organizations and employees in dealing with the blending of boundaries between work and life. However, our classification of determinants offers a valid basis for further investigations.

### 5.4 Limitations and conclusion

The evaluated studies offered valuable insights into the diverse and expansive body of research on the integration of work and home/life. Overall, the research field has evolved from focusing on boundary characteristics to examining external and internal factors that impact boundary management, and finally, exploring the relationship between work and life. More recently, the COVID-19 pandemic has significantly impacted the research field of Work-Life-Blending. Remote work arrangements and other disruptions have compelled many individuals to blend work and personal life, prompting a renewed focus on this topic in the post-pandemic world and highlighting new characteristics of the future of work (Malhotra, 2021). The latest studies are exploring the impact of these changes on individuals and organizations and how they can successfully navigate the boundaries between work and home life (e.g., Adisa et al., 2022; de Araújo Vitória et al., 2022). Taken together, the research field of Work-Life-Blending has evolved significantly over the last decades, reflecting broader individual and organizational changes towards greater flexibility, autonomy, and integration between work and personal life.

With our article, we pursued three research goals: (1) to review the state of the art about the blending of work and life, (2) to define and clarify the concept of Work-Life-Blending, and (3) to develop a future agenda for further research in this field. Concerning our goals, we conducted a systematic literature review, synthesizing and discussing results. We have achieved the final selection of the research using previously defined criteria and prioritization.

In general, systematic reviews have limitations. We are aware that these limitations can affect the quality and reliability of our findings. For instance, questions arise such as: Have all relevant studies been included? Has the quality of all studies been thoroughly assessed? Were the results adequately evaluated and summarized, and was the heterogeneity of the included studies considered? (Petticrew and Roberts, 2006). We aimed to minimize the risk of these limitations by following the PRISMA-Guidelines (Liberati et al., 2009; Moher et al., 2009). When searching the literature, we used different keywords, which we have derived inductively. However, we have not verified or expanded the keywords (e.g., through expert interviews) and might have missed further important keywords. In addition, we should have carried out an iterative process in the further course of the search.

One potential limitation may arise from the choice of databases. However, our selection of two databases (Business Source Premier and PsycARTICLES) is robust and well-suited to our research goals as they offer comprehensive coverage of relevant journals and provide a strong foundation for our investigation. Business Source Premier focuses on economic topics, while PsycARTICLES specializes in psychological topics. Business Source Premier and PsycARTICLES are large databases with extensive resources that provided sufficient output (4,934 studies and 1,400 studies after removing duplicates) for our research aim.

When screening the literature, we restricted our results to articles in English and German; however, we only excluded five articles in a different language. We screened 1,400 records based on title and abstract. Using our defined exclusion criteria, we condensed the results to 161 records gradually. With a view to our research goal, however, it was important to code the results in terms of their holistic and generic impact and to include them accordingly. We executed the coding at our discretion based on the criteria of *Antecedents*, *Processes*, and *Outcomes*. We consider this to be a limitation. However, as the interrater reliability was 43.52 per cent, we agreed on our final prioritisation.

In conducting this review, we chose the work/family border theory by Clark (2000) as our theoretical framework. Aligning with Rincy and Panchanatham (2014), we believe that Clark’s theory provides a structured approach to organizing relevant factors, which serves as a solid foundation for our review. While the theory is dated back to 2000, we recognize that work-life dynamics have evolved since then. Nevertheless, by utilising Clark’s theory as a starting point, we assume that the key concepts still hold relevance in contemporary work-life research. Furthermore, we are aware of the theory’s primary focus on how individuals manage and negotiate the boundaries between their work and family environments and on achieving a balance between work and life, while our review strives to maintain a neutral point of view and consider various dimensions and categories of the Work-Life-Blending.

In terms of methodologies, the studies included in our review encompass a variety of research approaches, including surveys, interviews, and experience-sampling studies. This diverse range of methodologies contributes to a comprehensive understanding of Work-Life-Blending, enabling a more nuanced analysis of boundary management practices among individuals in different work and family contexts. However, some studies have small sample sizes, such as Leppäkumpu and Sivunen (2023) with *N* = 32 informants, which could affect the generalizability of their results.

Regarding the participants and target groups of the studies, it is essential to note that the included studies involve various populations, including remote workers, subordinates and their spouses, alumni from business schools, employees in different industries, and others. The diversity of target groups may offer valuable insights into specific contexts and sectors. Additionally, some articles focus on individual characteristics and strategies for managing the work-life interface. The use of different methodologies allows for a comprehensive exploration of individual perspectives. However, self-report measures in survey-based studies may introduce response biases. Wan et al. (2022) utilize a survey-based approach to investigate the preferences of dual-earner couples for boundary segmentation and its impact on work–family conflict. While the study provides insights into the interactions between partners in Work-Life-Blending, the use of self-report measures may affect objectivity.

While we acknowledge that some of our original goals could not be fully achieved due to the evolving nature of the topic (particularly in light of the pandemic), we successfully defined and clarified the concept of Work-Life-Blending, which was a key aim of our study. Although we may have yet to be able to provide a complete summary of the latest state-of-the-art due to our holistic focus and due to the dynamics of COVID-19, our work provides a holistic foundation for future research and the development of a standardized measure.

The structure that emerged as part of the concept clarification enabled us to obtain an overview of the relevant factors and concept of Work-Life-Blending from a holistic perspective, considering the high degree of diversification in the research field. We are aware that our results are purely conceptual. However, with our research results, we were able to establish that there is no generic and holistic concept for the Work-Life-Blending and that many research strands are becoming increasingly diversified. Based on our generated categories Domains, Borders, Individual, and Interindividual, we have focused on relevant factors and findings to compile the status of the research field and developed a classification of determinants of Work-Life-Blending derived from our review. Although grounded in the extant literature, further research and the development of a conceptual model on this is necessary.

Overall, we hope our findings contribute to a comprehensive understanding of the complexities involved in Work-Life-Blending. The identified factors provide valuable insights for individuals, organizations, and researchers seeking to promote well-being. By recognizing the interplay of individual as well as interindividual factors, organizations can foster a supportive work environment that values work-life integration and empowers employees to manage their boundaries effectively. Therewith, we hope to support future research and contribute to the concept clarification, providing a foundation for future research to develop scales to assess the blending of work and life more holistically.

## Data availability statement

The original contributions presented in the study are included in the article/Supplementary material, further inquiries can be directed to the corresponding author/s.

## Author contributions

KS, SS, and CS: conceptualization, methodology, writing—original draft preparation, and writing—review and editing. KS: data search and visualization. KS and SS: data analysis. SS and CS: supervision. All authors have read and agreed to the published version of the manuscript.
